# Identification of Valid Reference Genes for the Normalization of RT-qPCR Expression Studies in Human Breast Cancer Cell Lines Treated with and without Transient Transfection

**DOI:** 10.1371/journal.pone.0117058

**Published:** 2015-01-24

**Authors:** Lin-Lin Liu, Hui Zhao, Teng-Fei Ma, Fei Ge, Ce-Shi Chen, Ya-Ping Zhang

**Affiliations:** 1 Laboratory for Conservation and Utilization of Bio-resource, Yunnan University, Kunming, China; 2 Department of Endocrine Surgery, The First Affiliated Hospital of Chongqing Medical University, Chongqing, 400016, China; 3 Key Laboratory of Animal Models and Human Disease Mechanisms of Chinese Academy of Sciences & Yunnan Province, Kunming Institute of Zoology, Chinese Academy of Sciences, Kunming, Yunnan, China; 4 State Key Laboratory of Genetic Resources and Evolution, Kunming Institute of Zoology, Chinese Academy of Sciences, Kunming, China; INRS, CANADA

## Abstract

Reverse transcription-quantitative polymerase chain reaction (RT-qPCR) is a powerful technique for examining gene expression changes during tumorigenesis. Target gene expression is generally normalized by a stably expressed endogenous reference gene; however, reference gene expression may differ among tissues under various circumstances. Because no valid reference genes have been documented for human breast cancer cell lines containing different cancer subtypes treated with transient transfection, we identified appropriate and reliable reference genes from thirteen candidates in a panel of 10 normal and cancerous human breast cell lines under experimental conditions with/without transfection treatments with two transfection reagents. Reference gene expression stability was calculated using four algorithms (geNorm, NormFinder, BestKeeper and comparative delta Ct), and the recommended comprehensive ranking was provided using geometric means of the ranking values using the RefFinder tool. GeNorm analysis revealed that two reference genes should be sufficient for all cases in this study. A stability analysis suggests that *18S rRNA-ACTB* is the best reference gene combination across all cell lines; *ACTB-GAPDH* is best for basal breast cancer cell lines; and *HSPCB-ACTB* is best for ER^+^ breast cancer cells. After transfection, the stability ranking of the reference gene fluctuated, especially with Lipofectamine 2000 transfection reagent in two subtypes of basal and ER^+^ breast cell lines. Comparisons of relative target gene (*HER2*) expression revealed different expressional patterns depending on the reference genes used for normalization. We suggest that identifying the most stable and suitable reference genes is critical for studying specific cell lines under certain circumstances.

## Introduction

Worldwide, breast cancer is the most common malignancy in females and accounts for approximately 30% of all cancers diagnosed [1]. Breast cancer is a complex and heterogeneous disease that can be classified into at least four subtypes: luminal A, luminal B, HER2 and basal cancers [2]. Each subtype has a different prognosis and treatment response [3], so it is crucial to develop robust biomarkers and reliable assays to detect, diagnose and treat specific breast cancers. Tumorigenesis is associated with gene expression changes; therefore, gene expression profiling is a common aspect of breast cancer research and drug treatment practices [4].

Reverse transcription-quantitative polymerase chain reaction (RT-qPCR) is a powerful technique for confirming gene expression differences or measuring transcript abundance due to its sensitivity, reproducibility, simplicity and high-throughput [5,6]. With this assay, the common method is normalization of gene expression using an endogenous reference gene. Ideal reference genes should be sufficiently abundant and have stable expression across different tissues and cell lines under different experimental conditions, but the ideal and universal reference gene does not exist in practice [7]. Inaccurate normalization can cause inadequate quantification and incorrect conclusions [7,8]. Currently, several mathematical approaches including geNorm [7], NormFinder [9], Bestkeeper [10] and comparative delta Ct [11] have been developed to assist appropriate reference gene selection and geNorm provides a measure of the minimum optimal number of reference genes to normalize [7].

Using different established breast cancer cell lines with molecular profiles observed in breast carcinomas (Table 1), cancer phenotype studies can be undertaken [2] if the appropriate reference gene can be used to normalize a gene of interest in those cell lines. Studies have identified valid reference genes for normalization in breast tumor and normal tissues [12–17], but only two studies are available for evaluating reference genes for cell lines [13,18]. One study included 4 human breast cell lines of increasing metastatic potential, such as MCF-10A, MCF-10T, MCF-7 and MDA-MB-231 [18], and the other used included ER^+^ breast cancer lines—T47D, MCF-7 and BrCa-MZ-01 [13]. Thus, more work is required to identify additional reference genes for more human breast cancer cell subtypes.

**Table 1:** Molecular classification of human normal and breast cancer cell lines.

**Classification**	**Immunoprofile**	**Cell lines in this study**
Luminal A	ER^+^, PR^+/−^, HER2^−^	MCF-7, T47D, HCC1500
Luminal B	ER^+^, PR^+/−^, HER2^+^	BT474
Basal	ER^−^, PR^−^, HER2^−^	HCC1806, SUM149PT, HCC1937, MDA-MB-231
HER2	ER^−^, PR^−^, HER2^+^	SKBR3
Normal	ER^−^, PR^−^, HER2^−^	MCF-10A

The application of transfection, used to introduce a gene of interest into a cell, is an important tool during molecular and cellular research. Two transfection approaches to introduce a gene of interest into a cell include transfection reagents (usually, liposome-based) and electroporation. These technologies are useful but the transfection reagents are cytotoxic [19] as documented by Jacobsen’s group who suggested that different transfection reagents give different transcriptional effects, regardless of the presence or absence of the gene of interest. Whether transient transfection using different transfection reagents can influence reference gene expression is unclear. Thus, we identified the most stable reference genes for normalization across human breast cell lines from different cancer subtypes with or without transient transfection. First, we searched the literature to select 13 genes (Table 2) to be candidate reference genes. A panel of 10 cell lines containing 5 cancer subtypes (Table 1) was either transiently transfected or not transfected with a control vector using either Lipofectamine 2000 or X-tremeGENE HP. Then reference gene expression stability was measured using 4 algorithms. Finally, the expression levels of target gene, epidermal growth factor receptor 2 (*HER2*), using the most/least stable and most used reference gene *GAPDH*, to validate the selection of candidate reference genes.

**Table 2:** Information on reference genes used in this study.

**Gene symbol**	**Gene name**	**Molecular function**	**Accession number**	**Chromosomal Localization**
*18S rRNA*	18S ribosomal RNA	Ribosome subunit, translation	NR_003286	ChrUn^a^
*ACTB*	β-Actin	Cytoskeletion	NM_001101	7p22-p12
*B2M*	β-2-microglobulin	Major histocompatibility complex	NM_004048	15q21-q22
*GAPDH*	Glyceraldehyde-3-phosphate dehydrogenase	Glycolysis	NM_002046	12p13.31
*HMBS*	Hydroxymethylbilane synthase	Porphyrin metabolism	NM_000190.3	11q23
*HPRT1*	Hypoxanthine phosphoribosyl-transferase 1	Generation of purine nucleotides	NM_000194	Xq26.1
*HSPCB*	Heat shock protein 90kDa alpha	Signal transduction	NM_007355	6p12
*PPIA*	Peptidylprolyl isomerase A	Protein folding	NM_021130	7p13
*PUM1*	Pumilio homolog	RNA bingding translation factor	NM_001020658.1	1p35.2
*RPS13*	ribosomal protein S13	Ribosome subunit	NM_001017	11p15.1
*SDHA*	Succinate dehydrogenase complex, subunit A	Glycolysis	NM_004168	5p15
*TBP*	TATA box binding protein	Transcription initiation	NM_003194	6q27
*YWHAZ*	Tyrosine 3-monooxygenase	Signal transduction	NM_003406	8q23.1

^a^ *Homo sapiens* unplaced genomic contig, GRCh37.p5.

## Materials and Methods

### Selection of reference genes

A total of 13 candidate reference genes were selected from a search of the relevant literature, particularly relating to reference genes previously identified in breast cancer. As can be seen in Table 2, the candidate genes ranged from traditional, commonly used reference genes such as *GAPDH* to less well known genes such as *HSPCB*, spanning a range of cellular functions. Among these candidate genes, *18S rRNA, ACTB, HPRT1, HSPCB, PPIA, PUM1, RPS13, SDHA* and *TBP* genes were reported to be optimal reference genes for normalization in breast cancer tumor and normal tissues [12–18,20]. In addition, the *B2M* gene was identified as a valid reference gene for expression studies in human colorectal tumor tissues [21] and in human stomach cancer [8]. The other 3 *GAPDH, HMBS* and *YWHAZ* genes are commonly used reference genes.

### Breast cell lines and culture conditions

One normal and nine breast cancer cell lines of four subtypes were used (Table 1). Cell lines were from American Type Culture Collection (ATCC, www.atcc.org). HCC1937, HCC1806, HCC1500, BT474 and T47D cells were cultured in RPMI1640 media with 5% fetal bovine serum, 4.5 g/L glucose (Amresco), 1 mM sodium pyruvate, 10 mM HEPES (Life technologies), 1.5 g/L sodium bicarbonate, 100 units/mL penicillin, 100 μg/mL streptomycin. MDA-MB-231 and SKBR3 cell lines were cultured in Dulbecco’s modified Eagle’s medium containing 10% fetal bovine serum, 100 units/mL penicillin and 100 μg/mL streptomycin. SUM149PT cells were cultured in Ham’s F12 containing 5 μg/mL insulin, 1 μg/mL hydrocortisone (Sigma), 10 mM HEPES, 100 units/mL penicillin and 100 μg/mL streptomycin. MCF7 cells were cultured in minimal essential medium containing 5% fetal bovine serum, 0.01 mg/mL insulin (Wanbang), 1 mM sodium pyruvate, 0.1 mM non-essential amino acids, 1.5 g/L sodium bicarbonate, 100 units/mL penicillin and 100 μg/mL streptomycin. The immortalized breast epithelial cell line MCF-10A was maintained in Dulbecco’s modified Eagle’s medium/Ham’s F-12 50/50 medium supplemented with 5% horse serum (Life technologies), 0.5 g/mL hydrocortisone, 10 g/mL insulin, 20 ng/mL epidermal growth factor (Sigma), 0.1 g/mL cholera enterotoxin (Wanbang), 100 units/mL penicillin, 100 μg/mL streptomycin and 2 mM L-glutamine. Cells were maintained in a humidified atmosphere with 5% CO_2_ at 37°C.

### Transfection treatments

The most frequently used expression vector pcDNA 3.1/myc-His(-) (Life technologies) was used as a control vector. One day prior to transfection, each cell line was placed in 6-well plates to a confluency of 70–90%, and then was transfected or was not transfected with vector using Lipofectamine 2000 Reagent (Life technologies) or X-tremeGENE HP DNA Transfection Reagent (Roche). Plasmid quantities and transfection reagents were 2.5 μg and 7.5 μL for Lipofectamine 2000 reagent, or 2 μg and 4 μL for X-tremeGENE HP DNA transfection reagent, respectively. After incubation for 48 h, cells were lysed by adding TRIzol LS Reagent (Invitrogen) directly. These experiments were performed in duplicate.

### RNA extraction and cDNA synthesis

Total RNA extraction including DNase treatment with RNase-free DNase I set (TianGen) was carried out using the RNeasy Mini Kit (Qiagen) according to the manufacturer’s instructions. Extracted RNAs were quantified by NanoDrop 2000 Spectrophotometer (Thermo Fisher Scientific), and the absorbance ratio at 260/280 and 260/230 were measured to assure RNA purity. RNA samples were then assessed with an RNA 6000 Nano kit (Agilent Technologies) using the Agilent 2100 electrophoresis Bioanalyzer (Agilent Technologies) to obtain an RNA integrity number (RIN). A threshold RIN value of 7 was applied, below which samples were excluded from analysis.

Total RNA (2 μg) was reverse-transcribed using the PrimeScript RT reagent Kit (Takara Biotechnology) in a total volume of 40 μL according to the manufacturer’s instructions. The RT primer Mix contained both oligo dT and random primers to obtain a maximum number of cDNA transcripts.

### Quantitative polymerase chain reaction (qPCR)

qPCR was performed on 13 putative reference genes and one target gene of *HER2*. Their characteristics are summarized in Table 3. Primer pair sequences were selected from the literature or were designed using primer Express 3.0 software. Each primer set was confirmed to be specific to its targeting gene with no homology to other sequences at UCSC’s human genome browser (http://genome.ucsc.edu). PCR products were further cloned into PMD18-T vector (Takara Biotechnology) and sequenced for verification using an ABI PRISM 3730 DNA sequencer according to the manufacturer’s recommendations (Applied Biosystems).

**Table 3:** Primers for 13 reference genes and a target gene.

**Gene**	**Forward and Reverse Primer (5′ → 3′)**	**Product (bp)**	**R^2^**	**E** ^a^ ** (%)**	**Intron spanning**	**Reference**
*18S rRNA*	GGATGTAAAGGATGGAAAATACA	72	0.994	96	NA^b^	This study
	TCCAGGTCTTCACGGAGCTTGTT					
*ACTB*	TGACGTGGACATCCGCAAAG	205	0.996	107	Yes	This study
	CTGGAAGGTGGACAGCGAGG					
*B2M*	CACCCCCACTGAAAAAGATG	167	0.996	93	Yes	[33]
	ATATTAAAAAGCAAGCAAGCAGAA					
*GAPDH*	GACAGTCAGCCGCATCTTCT	127	0.993	98	Yes	[33]
	TTAAAAGCAGCCCTGGTGAC					
*HMBS*	CTGTTTACCAAGGAGCTGGAAC	100	0.992	110	Yes	[34]
	TGAAGCCAGGAGGAAGCA					
*HPRT1*	GACCAGTCAACAGGGGACAT	132	0.995	109	Yes	[35]
	CCTGACCAAGGAAAGCAAAG					
*HSPCB*	TCTGGGTATCGGAAAGCAAGCC	80	0.998	99	Yes	[20]
	GTGCACTTCCTCAGGCATCTTG					
*PPIA*	AGACAAGGTCCCAAAGAC	118	0.996	90	Yes	[20]
	ACCACCCTGACACATAAA					
*PUM1*	CAGGCTGCCTACCAACTCAT	211	0.995	95	Yes	[35]
	GTTCCCGAACCATCTCATTC					
*RPS13*	CGAAAGCATCTTGAGAGGAACA	87	0.992	92	Yes	[20]
	TCGAGCCAAACGGTGAATC					
*SDHA*	TGGTTGTCTTTGGTCGGG	85	0.992	106	Yes	[36]
	GCGTTTGGTTTAATTGGAGGG					
*TBP*	GAGAGTTCTGGGATTGTACCG	143	0.995	106	Yes	[36]
	ATCCTCATGATTACCGCAGC					
*YWHAZ*	ATGCAACCAACACATCCTATC	178	0.995	104	Yes	[34]
	GCATTATTAGCGTGCTGTCTT					
*HER2*	TGACACCTAGCGGAGCGA	184	0.994	97	Yes	This study
	GGGGATGTGTTTTCCCTCAA					

^a^Efficiency value.

^b^Not available.

RT-qPCR reactions were conducted in a 96-well plate using ABI PRISM 7000 Real-Time system (Applied Biosystems). Each reaction was performed in triplicate and in a 20 μL volume containing 1× SYBR Premix Ex Taq II (Takara Biotechnology), 50 nM of each primer and 0.8 μL cDNA. The cycling conditions were as follows: 95°C for 10 sec, followed by 40 cycles of 95°C for 5 sec and 61°C for 31 sec. PCR reaction specificity was confirmed by DNA melting curve analysis and gel electrophoresis of product. Each experiment included a no-template control and a cDNA standard curve for each gene. The dynamic range of the standard curve spanned seven orders of each gene cDNA from the HCC1806 cell line. The reaction efficiency (E) of each gene was calculated according to the formula E = [10^(−1/slope)^-1].

### Data analysis

Candidate reference gene stability was evaluated using a web-based comprehensive tool RefFinder (http://www.leonxie.com/referencegene.php), which includes the four most commonly used approaches such as geNorm [7], NormFinder [9], BestKeeper [10] and the comparative delta Ct [11]. First, geNorm calculates M for each candidate gene based on pairwise comparisons of variable. Genes with smaller M are usually associated with high expression stability. GeNorm also calculates the optimal number of reference genes for gene expression analysis [7]. 2) NormFinder estimates the overall variation of gene expression for each candidate gene and delivers a stability value, not only identifying the most stable reference genes but also the best overall control gene [9]. 3) BestKeeper uses pair-wise correlations [10]; and 4) the comparative delta Ct method ranks reference gene stability according to reproducibility of gene expression differences [11]. Based on the ranks observed from each program, RefFinder assigns an appropriate weight to an individual gene and calculated the geometric mean of their weights for the overall final ranking. Comparisons of means were carried out using a Student’s test with the SPSS 22.0 software (IBM).

### Literature review

A PubMed database (http://www.ncbi.nlm.nih.gov/pubmed/) review was performed using the key words: ‘reference genes’ OR ‘housekeeping genes’ AND ‘qPCR’ OR ‘quantitative PCR’ AND ‘breast cancer cell’ between January 2000 and June 2014.

## Results

### Quality and integrity of RNA samples

To assure sample quality and integrity, RNA was extracted from fresh cells and treated with DNaseI to avoid amplification from residual genomic DNA. Absorbance ratios 260/280 nm and 260/230 nm, averaged (mean ± standard deviation) over all 10 cell lines, were 2.065±0.052 and 2.075±0.114, respectively (S1 Table). Moreover, the RIN ranged from 8.8–10 (S1 Table). These data indicated that our samples were of sufficient total RNA quality and integrity.

### Amplification specificity and primer optimization

Primers for 13 reference genes and one target gene were highly specific, yielding single bands when the PCR products were separated on an agarose gel, and single peaks in dissociation curves of qPCR reactions (data not shown). Gene-specific amplifications were further confirmed by sequencing analysis. Obtained reference genes and target gene sequencing results were identical to the reference sequences. Thus, each qPCR reaction was specific.

PCR reaction efficiency of each gene was measured with 10-fold serial dilutions of cDNA of each gene (S1 Fig.). Efficiency values (E) and correlation coefficients (R^2^) for each primer pair are shown in Table 3. For each candidate reference gene, R^2^ was not less 0.992 and E values were 90–110% (inclusive).

### Candidate reference gene expression

**Figure 1:**
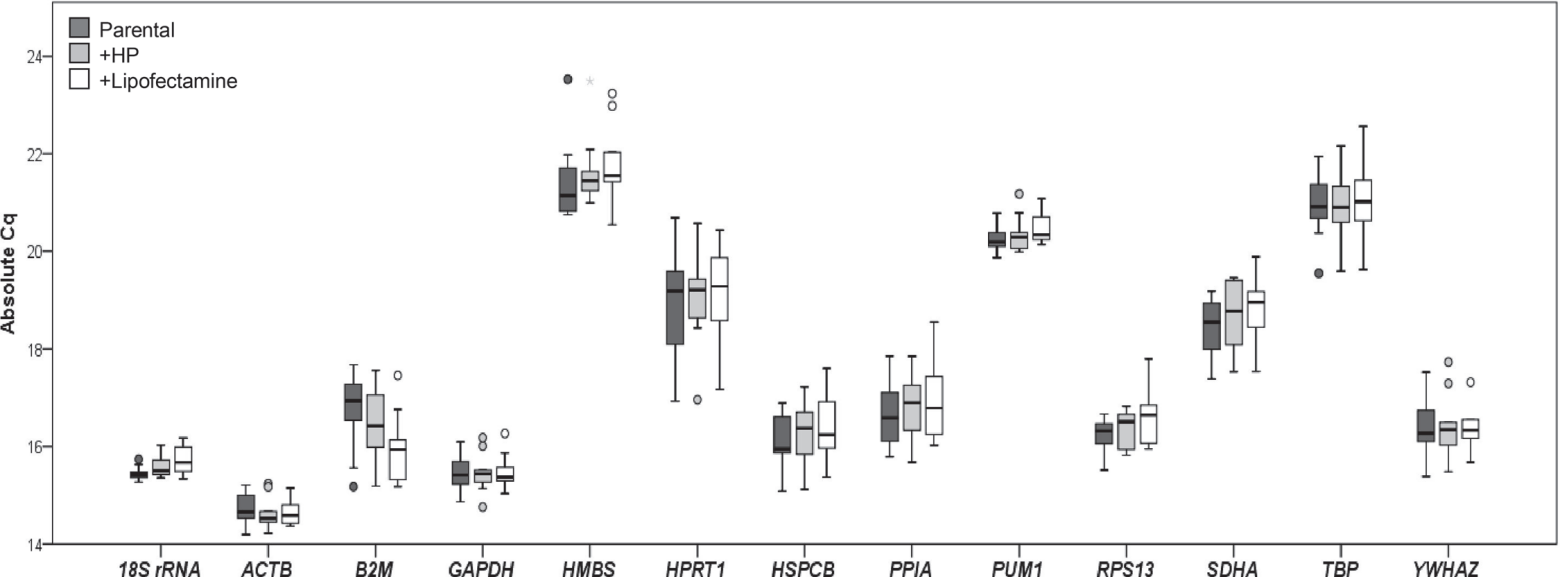
Box plot of absolute Cq values for each reference gene. Expression of selected reference genes in 10 human breast cells, which were not treated (Parental), or were transiently transfected with X-tremeGENE HP DNA transfection reagent (+HP) or with Lipofectamine 2000 transfection reagent (+Lipofectamine) as well as with plasmid displayed as Cq. The median is indicated by a line in each box, which in turn represents the 25^th^ and 75^th^ percentile. Whiskers indicate the 10/90 percentile ranges, circles represent potential outliers.

With RT-qPCR experiments across 10 cell lines with/without transfection treatments, we obtained absolute Cq values (based on the nomenclature and MIQE guidelines: the quantification cycle (Cq) is preferred to the threshold cycle (Ct)) for each gene under different conditions). The median, 25^th^ and 75^th^ percentiles, and range of Cq values for each gene are presented in Fig. 1. The absolute expression of the 13 reference genes were observed spanning from the most abundant (*ACTB*, mean Cq 14.65) to the least abundant genes (*HMBS*, mean Cq 21.60), indicating their moderate abundance in the observed samples. By pair-wise t-test, significant differences in variance were observed between candidates (P < 0.001), with genes such as *18S rRNA* and *ACTB* genes having less variance than others (*B2M* and *PPIA*, Fig. 1). Moreover, the intra-run coefficient variation (CV) for each candidate reference gene ranged from 1.55% for *18S rRNA* to 5.06% for *HPRT1* with a mean CV for all genes of 3.28%, in which CV was computed using the ratio of SD and the average. In addition, within each transiently transfected cell line, the change of Cq for reference genes spanned from 0.00142 for *GAPDH* untreated and treated with X-tremeGENE HP transfection reagent to 1.029 for *B2M* untreated and treated with Lipofectamine 2000. The data suggest that *ACTB* expression was considerably stable, and there was more variation for *B2M* with Lipofectamine 2000 transfection treatment.

### Identification of optimal reference genes

To identify the most stable reference genes in tested breast normal and cancer cell lines, we used four analyses: geNorm, NormFinder, BestKeeper and comparative delta Ct. Genes with a lower stability value or an average STDEV are usually associated with high expression stability. In addition, the RefFinder program provides the recommended comprehensive ranking by calculating geometric means from ranking orders analyzed by the four algorithms. Table 4 shows that both comparative delta Ct and NormFinder suggested that *ACTB* and *18S rRNA* were the two most stable reference genes, and geNorm and BestKeeper identified *18S rRNA* and *PUM1* as the best reference genes across the 10 cell lines studied. Consequently, *18S rRNA, ACTB* and *PUM1* were identified to be the 3 most stably expressed reference genes by RefFinder. All tools ranked *HPRT1* as the least suitable reference gene across all cell lines. Next, we evaluated the stability of reference genes for basal (HCC1806, SUM149PT, HCC1937 and MDA-MB-231) and ER^+^ breast cancer cell lines (MCF-7, T47D, HCC1500 and BT474). *ACTB* and *GAPDH* were the two most stable reference genes in basal breast cancer cells, and *HSPCB* and *ACTB* were best in ER^+^ breast cancer cell lines (Table 5). Interestingly, *HSPCB* was not a stable reference gene in basal breast cancer cells (ranking 10) or across all 10 cell lines (ranking 7), whereas it was the most stable reference gene in ER^+^ breast cancer cells (Table 5) indicating that the stability of the reference gene depends on the cell lines.

**Table 4:** Stability ranking of 13 reference genes analyzed by five algorithms across all cell lines.

**Delta Ct**		**BestKeeper**		**NormFinder**		**geNorm**		**RefFinder**	
**Gene in ranking order**	**Average of STDEV**	**Gene in ranking order**	**Average of STDEV**	**Gene in ranking order**	**Stability value**	**Gene in ranking order**	**Stability value**	**Gene in ranking order**	**Geomean of ranking values**
*ACTB*	0.53	*18S rRNA*	0.10	*ACTB*	0.056	*18S rRNA*	0.267	*18S rRNA*	1.41
*18S rRNA*	0.59	*PUM1*	0.22	*18S rRNA*	0.301	*PUM1*	0.267	*ACTB*	1.73
*YWHAZ*	0.61	*ACTB*	0.23	*YWHAZ*	0.312	*ACTB*	0.336	*PUM1*	2.51
*PUM1*	0.61	*RPS13*	0.24	*GAPDH*	0.347	*GAPDH*	0.368	*GAPDH*	4.47
*GAPDH*	0.62	*GAPDH*	0.28	*PUM1*	0.350	*RPS13*	0.391	*YWHAZ*	4.88
*HSPCB*	0.63	*SDHA*	0.45	*HSPCB*	0.360	*HSPCB*	0.450	*RPS13*	5.60
*RPS13*	0.69	*HSPCB*	0.48	*RPS13*	0.483	*YWHAZ*	0.478	*HSPCB*	6.24
*TBP*	0.71	*TBP*	0.48	*TBP*	0.501	*TBP*	0.509	*TBP*	8.00
*SDHA*	0.76	*YWHAZ*	0.50	*SDHA*	0.567	*SDHA*	0.544	*SDHA*	8.13
*HMBS*	0.79	*PPIA*	0.54	*HMBS*	0.618	*HMBS*	0.582	*HMBS*	10.24
*PPIA*	0.88	*HMBS*	0.59	*PPIA*	0.739	*PPIA*	0.622	*PPIA*	10.74
*B2M*	0.95	*B2M*	0.61	*B2M*	0.830	*B2M*	0.673	*B2M*	12.00
*HPRT1*	0.98	*HPRT1*	0.79	*HPRT1*	0.863	*HPRT1*	0.719	*HPRT1*	13.00

**Table 5:** Ranking of reference genes in order of stability treated with or without transfection.

**All breast normal and cancer cells**			**Basal breast cancer cells**			**ER^+^ breast cancer cells**		
**Parental**	**+HP**	**+Lipofectamine**	**Parental**	**+HP**	**+Lipofectamine**	**Parental**	**+HP**	**+Lipofectamine**
*18S rRNA*	*18S rRNA*	*ACTB*	*ACTB*	*ACTB*	*PUM1*	*HSPCB*	*18S rRNA*	*RPS13*
*ACTB*	*ACTB*	*18S rRNA*	*GAPDH*	*GAPDH*	*ACTB*	*ACTB*	*ACTB*	*18S rRNA*
*PUM1*	*PUM1*	*PUM1*	*HMBS*	*PUM1*	*18S rRNA*	*18S rRNA*	*RPS13*	*ACTB*
*GAPDH*	*YWHAZ*	*YWHAZ*	*PUM1*	*HMBS*	*HMBS*	*RPS13*	*HSPCB*	*HSPCB*
*YWHAZ*	*GAPDH*	*GAPDH*	*18S rRNA*	*18S rRNA*	*GAPDH*	*B2M*	*YWHAZ*	*YWHAZ*
*RPS13*	*RPS13*	*RPS13*	*PPIA*	*PPIA*	*SDHA*	*HMBS*	*HMBS*	*TBP*
*HSPCB*	*HSPCB*	*HSPCB*	*RPS13*	*SDHA*	*YWHAZ*	*TBP*	*TBP*	*GAPDH*
*TBP*	*TBP*	*TBP*	*SDHA*	*RPS13*	*PPIA*	*YWHAZ*	*GAPDH*	*HMBS*
*SDHA*	*HMBS*	*SDHA*	*YWHAZ*	*YWHAZ*	*RPS13*	*GAPDH*	*PUM1*	*PUM1*
*HMBS*	*SDHA*	*HMBS*	*HSPCB*	*HSPCB*	*HSPCB*	*PUM1*	*PPIA*	*PPIA*
*PPIA*	*B2M*	*PPIA*	*TBP*	*TBP*	*TBP*	*PPIA*	*B2M*	*SDHA*
*B2M*	*PPIA*	*HPRT1*	*B2M*	*B2M*	*B2M*	*SDHA*	*SDHA*	*HPRT1*
*HPRT1*	*HPRT1*	*B2M*	*HPRT1*	*HPRT1*	*HPRT1*	*HPRT1*	*HPRT1*	*B2M*

Cells were not treated (Parental), or were transiently transfected with X-tremeGENE HP DNA transfection reagent (+HP) or with Lipofectamine 2000 transfection reagent (+Lipofectamine) as well as with plasmid.

### Transfection treatment effects on reference gene stability

A suitable reference gene should not vary in expression between cells with and without treatments. Thus, we studied gene expression in the presence of Lipofectamine 2000 or HP transfection reagents in human breast cell lines. Reference gene stability was ranked by RefFinder (Table 5) and data indicate that stability ranking did not substantially change with/without transfection across all cell lines. The 3 most stable genes were *18S rRNA, ACTB* and *PUM1*, and the 3 least stable genes were *PPIA, B2M* and *HPRT1*. However, after transfection treatments reference gene stability fluctuated in basal and ER^+^ breast cell lines. Stability rankings of *HSPCB* changed from the 1^st^ position without treatment to the 4^th^ position with treatments in ER^+^ breast cancer cells. For *GAPDH*, similar findings were noted in basal breast cancer cells.

### Optimal reference gene numbers

**Figure 2:**
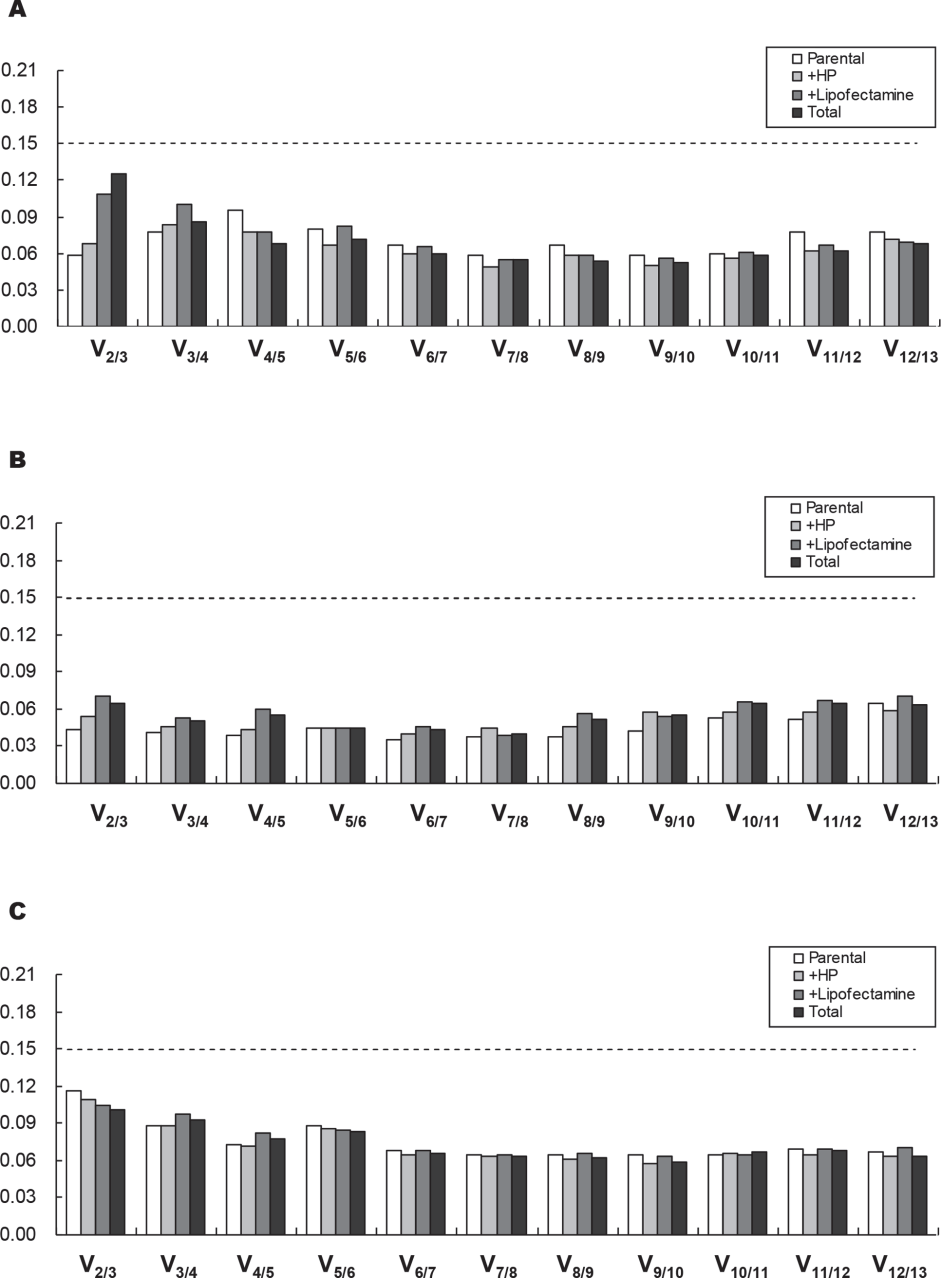
Determination of the optimal number of reference genes for normalization. Pair-wise variation value (Vn/n+1) was generated by geNorm analysis from basal breast cancer cell lines (A), ER^+^ breast cancer cell lines (B), and all 10 breast cell lines (C). The dash line indicates the cut-off value of 0.15.

To determine the optimal number of genes required for geometric mean normalization, geNorm was used to calculate the pairwise variation (V_n_/V_n+1_) between sequential normalization factors (NF) (NF_n_/NF_n+1_). A recommended threshold of 0.15 for the pairwise variation [7] was adopted as a cut-off for reference gene inclusion. Data indicate that a value of V_2/3_ fell below the threshold of 0.15 in basal breast cancer cell lines (Fig. 2A), ER^+^ breast cancer cell lines (Fig. 2B), and across all breast cancer cell lines (Fig. 2C), regardless of transfection treatments. Therefore, the use of two reference genes should be sufficient in this study. For normalization of cells without transfection treatments, *18S rRNA* and *ACTB* would be appropriate across all cells, and *GAPDH* and *ACTB* would be appropriate in basal breast cancer cells. *HSPCB* and *ACTB* would be best in ER^+^ breast cancer cells (Table 5). For data normalization of cells with transfection treatments, *18S rRNA* and *ACTB* should be used as valid reference genes across all cell lines (S2 Table).

### Effect of reference gene normalization on HER2 expression

**Figure 3:**
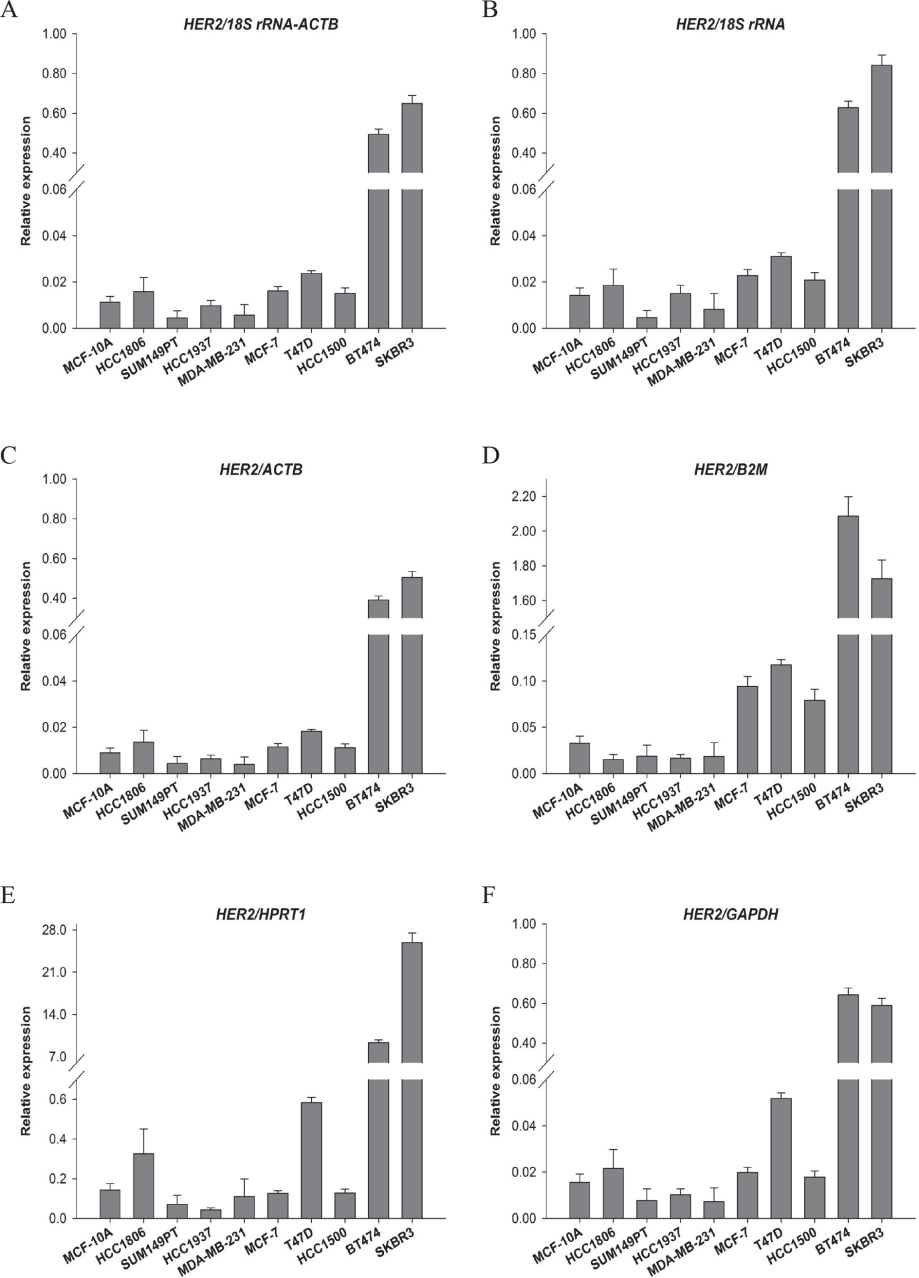
Relative quantification of *HER2* expression depends on different reference genes. Relative expression of *HER2* across all cell lines was normalized by the best combination (*18S rRNA-ACTB*) (A), by the most stable single gene *18S rRNA* (B) or *ACTB* (C), by the least stable single gene *B2M* (D) or *HPRT1* (E), and by the most used but not recommended gene *GAPDH* (F).

To measure the effect of reference gene normalization on gene expression data, we selected human *HER2* as a target gene. *HER2* is a member of the epidermal growth factor family, and is present in approximately 20–30% of breast cancer tumors. Importantly, *HER2* expression is associated with breast cancer pathophysiology and therapy [22]. As shown in Fig. 3, relative expression of *HER2* was calculated with 5 individual reference genes and the combination of two suitable reference genes using the 2^−∆∆Ct^ method. As predicted, a significant overexpression of *HER2* was present in HER2^+^ subtype cell lines, such as BT474 and SKBR3. *HER2* expression normalized by *18S rRNA* or *ACTB* as single reference gene and by the *18S rRNA*-*ACTB* combination was the optimal combination across all breast cell lines, and had similar high-low patterns (Fig. 3A-C). Normalization using the least stable reference genes *B2M* or *HPRT1* yielded unusual expressional patterns (Fig. 3D and E). In comparison, *HER2* expression reduced expression by *HPRT1* in HCC1937, MCF7 and HCC1500 cells (Fig. 3E) and by *B2M* in HCC1806 and SKBR3 cells (Fig. 3D). Additionally, stability of *GAPDH*, the most used reference gene, ranked 4^th^ across all breast cells in this study (Table 4). When normalization with *GAPDH*, relative *HER2* expression yielded contrasting expressional patterns for BT474 and SKBR3 cells (Fig. 3F) compared data in Fig. 3A. These results indicate that relative expression of *HER2* could be interpreted in different ways depending on the reference genes used for normalization.

## Discussion

Analysis of gene expression involved in cancer tumorigenesis and metastasis is key to tailoring individual treatments and RT-qPCR can assist with this. In RT-qPCR analysis, the RNA integrity is a critical first step in obtaining meaningful gene expression data, because an exhaustive analysis of the influence of RNA integrity offers different conclusions[23]. The RIN value is a useful tool for RNA integrity assessment. In general, a RIN value higher than 5 was considered to be good total RNA quality and higher than 8 as “perfect” total RNA for downstream application [23]. In this study, all RIN values exceeded 8.8, indicating that our total RNA samples were of sufficient quality. Secondly, primer selection is important [24], so we used previously reported primer pairs or designed primer sets to amplify an amplicon of 70–250 bp. Amplification is “independent” of RNA quality during the amplicon range [25]. Lastly, all primer pairs spanned neighboring exons except for the *18S rRNA* gene which is not an mRNA and this contributed to amplification efficiency and gave E values and R^2^ of studied reference and target genes ranging as depicted in Table 3.

We then selected 13 reference genes from the literature. Ten (*18S rRNA, ACTB, B2M, HPRT1, HSPCB, PPIA, PUM1, RPS13, SDHA* and *TBP*), were reported to be the most stable genes in several human tissues from breast, colon and stomach cancers [8,12–18,20–21]. In this study, these reference genes had moderate abundance with mean Cq values of 14–22, and high expression stability with low stability values (M) (< 0.8) according to geNorm analysis, below the default limit of M = 1.5 [7]. To identify the most suitable reference genes across 10 breast cell lines, expression stabilities of candidates were calculated using the four most frequently used algorithms. Inconsistent results were observed with these methods due the unique strategy of each technique to assess gene stability (Table 4, S2 Table). Therefore, we calculated the geomean of ranking values from the four methods and obtained a comprehensive ranking using RefFinder tool. Data show that *18S rRNA* and *ACTB* were the most stable reference genes across all investigated cell lines, regardless of transfection treatments (Table 4, S2 Table) and the literature supported this finding. *ACTB* was verified as one of the best combination genes for breast tumor and normal tissues [15,16]. Both *18S rRNA* and *ACTB* have been suggested to be suitable for normalization among a set of human breast cancer cell lines of increasing metastatic potential, but limitations do exist [18]. In contrast, *HPRT1* was identified as the least stable reference gene by all algorithms (Table 4), but it was reported as the single best reference gene in 80 normal and tumor samples [17]. An explanation for this may be that different sample resources between breast cell lines in our study and tissue samples used by others (colorectal, breast, prostate, skin and bladder tumor in work by de Kok’s group) [17].

*GAPDH* has been widely used as a reference gene in RT-qPCR analysis. However, *GAPDH* is reported to be involved in biological processes [26] and *GAPDH* expression is substantially increased in human cancers from the cervix, prostate, pancreas and lung [27–30], and in MCF7 cells treated with oestradiol [31]. Therefore, *GAPDH* has not been suggested to be a control RNA to study breast cancer [12,16,17,31]. We observed that *GAPDH* was not the most stable reference gene in our experiments as well (Table 4). Even so, *GAPDH* continues to be utilized as a normalizer in breast cancer and cell line studies with RT-qPCR. When a PubMed database review was performed, almost half of the publications indicated that *GAPDH* was used a single reference gene for normalization in gene expression analyses with qPCR technology. Particular attention should be paid to the selection of *GAPDH* as a reference gene in future studies.

To illustrate typical errors of data interpretation, we normalized the relative expression of *HER2* using different reference genes. Fig. 3 shows that there was a tendency for *HER2* expression to change depending on the reference gene. *HER2* expression normalized with the best single reference gene (*18S rRNA* or *ACTB*) or a combination of these indicated stable expression patterns across all cell lines (Fig. 3A-C). However, when normalizing to a gene (*GAPDH, B2M* or *HPRT1*) with unstable expression, the relative amount of target gene expression calculation caused erroneous conclusions (Fig. 3D-F). *HER2* expression normalized by the worst genes—*B2M* and *HPRT1*—was substantially increased in all investigated cells. Similarly, Warrington and coauthors verified that expression of genes normalized by frequently used reference genes could vary by 7- to 23-fold, depending on the cell type or tissue [32].

To assess the stabilities of reference genes affected by transfection, we performed negative control transfection using Lipofectamine 2000 or X-tremeGENE HP DNA transfection reagent and the expression vector. Data show that candidate stabilities were not influenced across all tested cells, but fluctuated significantly in ER^+^ breast cancer cells after transfection treatments (Table 5). Of note, Lipofectamine 2000 transfection reagent had the greatest effect on reference gene stability compared to X-tremeGENE HP DNA transfection reagent both in basal and ER+ cell lines (Table 5). Similar reports suggest that many transcripts were changed in the presence of Lipofectamine 2000, regardless of the presence/absence of the gene of interest [19]. This supports the suggestion that selecting the best transfection reagent along with the appropriate vector is necessary to ensure that most observed responses are biological effects of the target gene and not based on a particular transfection process used [19].

## Conclusions

To the best of our knowledge this is the first systematic identification of reference genes for qPCR studies in human breast cancer cell lines containing different cancer subtypes treated with transient transfection. We have validated two genes, *18S rRNA* and *ACTB* as control genes for RT-qPCR analysis of human breast cancer cell lines containing different subtypes using 4 different mathematical approaches. After transient transfection, reference genes can vary with the subtype of cell lines and therefore identifying the most stable and suitable reference genes is critical for studying specific cell lines under certain circumstances.

## Supporting Information

S1 Fig Standard curve for all primer pairs. (PDF)

S1 Table Quantity and integrity of total RNA. (PDF)

S2 Table Ranking reference genes based on the all five algorithms. Cells were not treated (Parental), or were transiently transfected with X-tremeGENE HP DNA transfection reagent (+HP) or with Lipofectamine 2000 transfection reagent (+Lipofectamine).
(XLS)
