# Supplementary figures and images for: Identification of Valid Reference Genes for the Normalization of RT-qPCR Expression Studies in Human Breast Cancer Cell Lines Treated with and without Transient Transfection
Source: PLoS One. 2015 Jan 24;10(1):e0117058. doi: 10.1371/journal.pone.0117058 (PMC4305315; doi:10.1371/journal.pone.0117058)

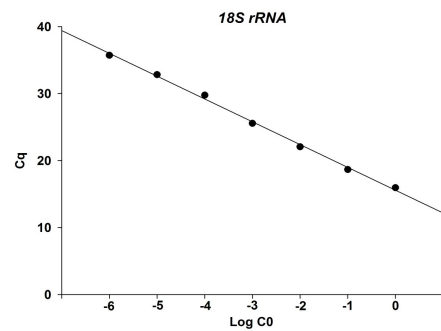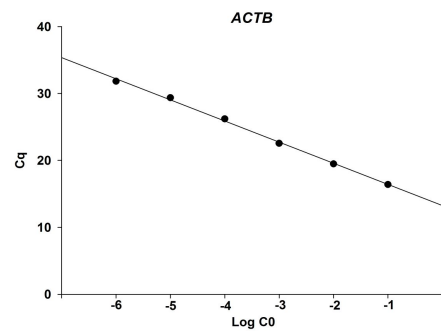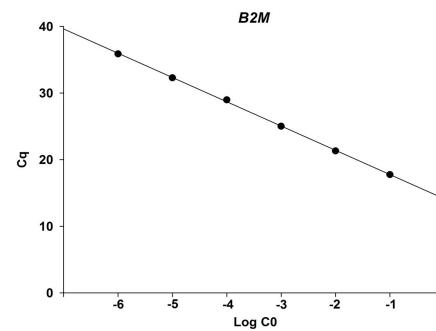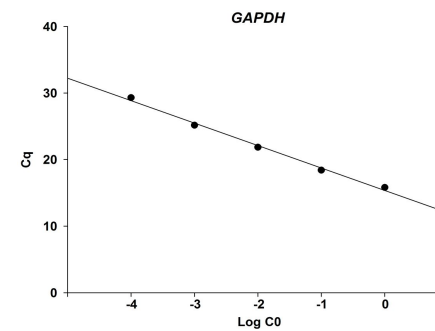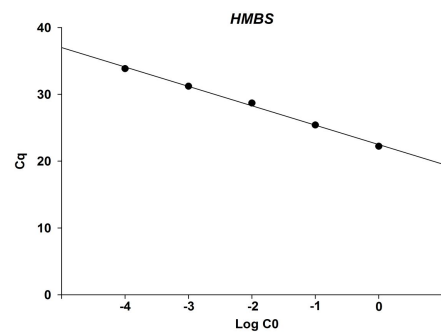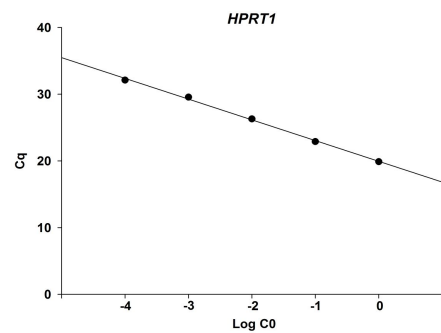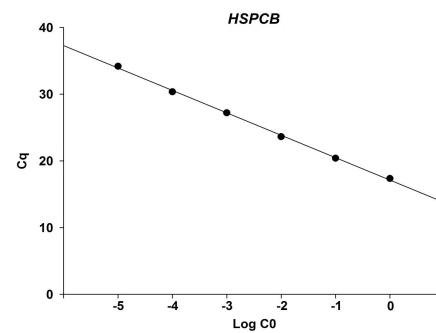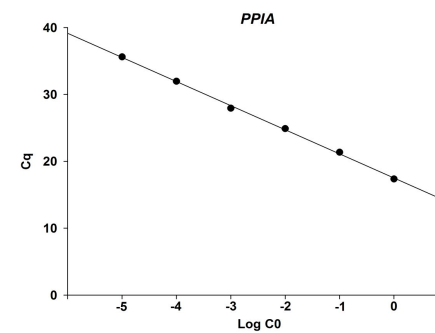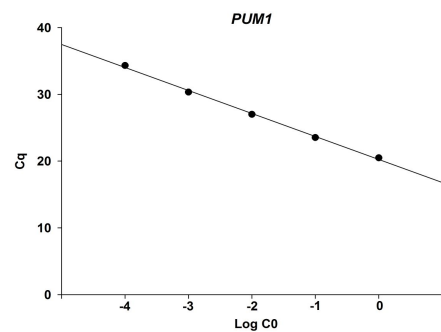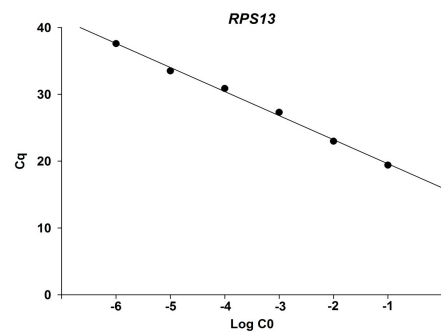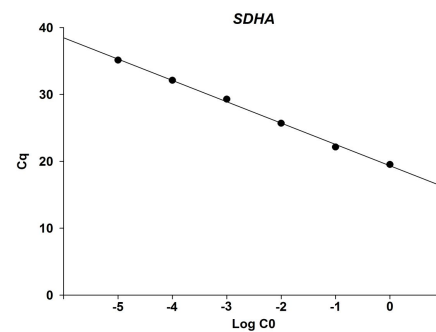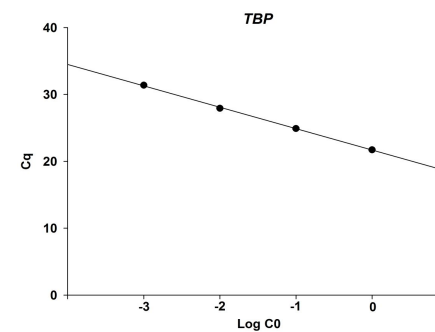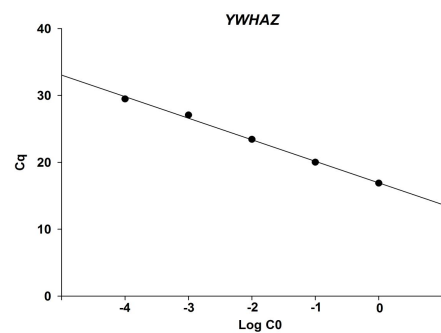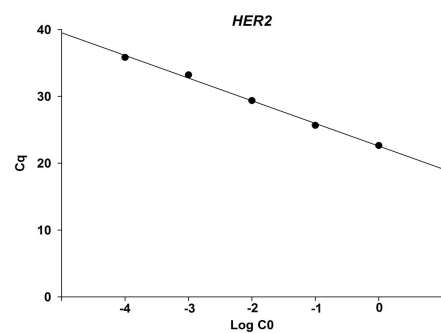

Supplement: S1 Fig — (PDF) [file pone.0117058.s001.pdf]
