# Supplementary material for: Identification of Valid Reference Genes for the Normalization of RT-qPCR Expression Studies in Human Breast Cancer Cell Lines Treated with and without Transient Transfection
Source: PLoS One. 2015 Jan 24;10(1):e0117058. doi: 10.1371/journal.pone.0117058 (PMC4305315; doi:10.1371/journal.pone.0117058)
Supplement: S1 Table — (PDF) [file pone.0117058.s002.pdf]

Table S1. Summarized results on RNA quality and integrity check in investigated cell lines.

Cell line	RNA conc. (ng/ul)	260/280	260/230	RIN	28S/18S
HCC1806	209.3	2.09	1.96	10	2.3
HCC1806+HP	196.7	2.10	2.01	10	2.1
HCC1806+lip2000	202.5	2.12	2.12	9.8	2.1
SUM149PT	231.7	2.09	2.15	9.4	2.1
SUM149PT+HP	190.9	2.09	2.12	8.8	1.9
SUM149PT+lip2000	229.7	2.08	2.12	9.6	2.0
SKBR3	241.0	2.11	1.95	9.9	1.8
SKBR3+HP	172.6	2.09	1.85	10	1.5
SKBR3+lip2000	267.2	2.11	1.89	9.7	1.8
BT474	284.5	2.13	2.18	10	1.9
BT474+HP	221.0	2.13	2.25	10	1.8
BT474+lip2000	196.3	2.14	2.23	9.7	1.9
MCF-10A	321.6	2.10	2.15	10	2.0
MCF-10A+HP	260.0	2.09	1.90	10	1.9
MCF-10A+lip2000	290.6	2.07	1.94	10	2.0
MCF-7	299.3	2.09	2.15	9.8	1.9
MCF-7+HP	227.9	2.08	2.16	9.6	1.9
MCF-7+lip2000	188.1	2.08	1.94	9.6	1.7
T47D	274.8	2.07	2.15	9.6	1.7
T47D+HP	198.8	2.08	1.97	9.6	1.8
T47D+lip2000	165.7	2.06	2.03	9.1	1.5
HCC1500	340.1	2.01	2.15	9.8	1.9
HCC1500+HP	175.6	2.02	2.16	9.5	1.5
HCC1500+lip2000	177.6	2.03	2.12	9.7	1.9
HCC1937	289.9	2.01	2.12	10	1.8
HCC1937+HP	290.0	2.02	2.13	10	1.8
HCC1937+lip2000	236.5	2.03	2.14	9.8	1.8
MDA-MB-231	240.8	2.02	2.12	9.8	2.1
MDA-MB-231+HP	228.8	2.01	2.13	9.6	1.9
MDA-MB-231+lip2000	222.6	2.03	2.15	9.6	1.9
